# Supplementary material for: Deletion of wfs1 Impairs Oligodendrocyte Precursor Cells Dorsal Distribution and Myelination Through the wfs1-hmgcs1 Axis in Zebrafish
Source: Biology (Basel). 2026 Aug 16;15(16):1402. doi: 10.3390/biology15161402 (PMC13510151; doi:10.3390/biology15161402)
Supplement: Supplementary file 1 [file biology-15-01402-s001.zip › File S1-Transcriptome sequencing data and differentially expressed genes (DEGs) in wfs1-- zebrafish at 5 dpf/2.KEGG/wfs1KOvsWT/ALL/wfs1KOvsWT.all_KEGGbar.pdf]

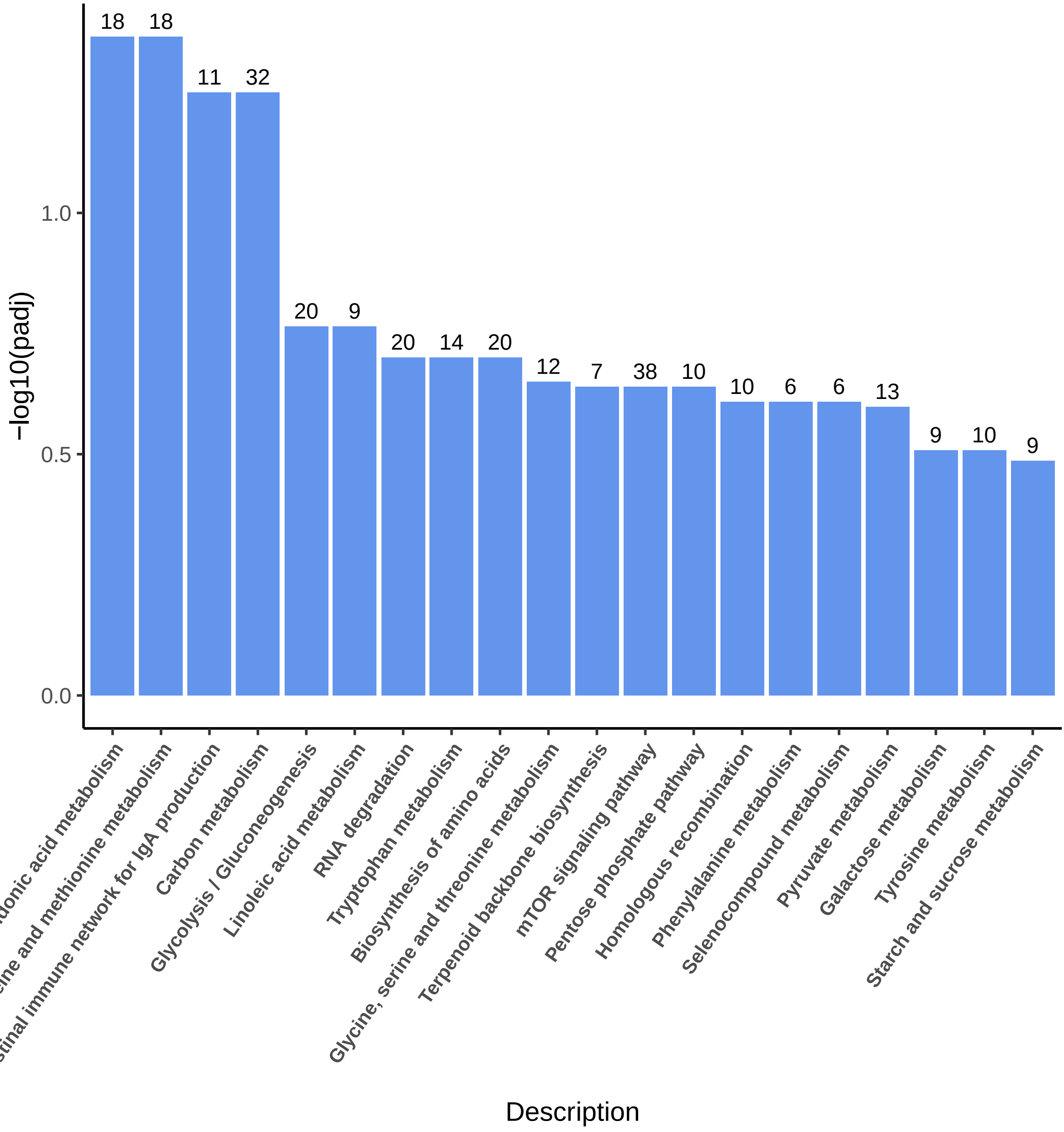

18
18
11
32
20
9
20
14
20
12
7
38
10
10
6
6
13
9
10
9
1.0
0.5
0.0
−log10(padj)
Arachidonic acid metabolism
Cysteine and methionine metabolism
Intestinal immune network for IgA production
Carbon metabolism
Glycolysis / Gluconeogenesis
Linoleic acid metabolism
RNA degradation
Tryptophan metabolism
Biosynthesis of amino acids
Glycine, serine and threonine metabolism
Terpenoid backbone biosynthesis
mTOR signaling pathway
Pentose phosphate pathway
Homologous recombination
Phenylalanine metabolism
Selenocompound metabolism
Pyruvate metabolism
Galactose metabolism
Tyrosine metabolism
Starch and sucrose metabolism
Description
